# Tks5 SH3 domains exhibit differential effects on invadopodia development

**DOI:** 10.1371/journal.pone.0227855

**Published:** 2020-01-30

**Authors:** Christina Daly, Brewer Logan, Joseph Breeyear, Kelley Whitaker, Maryam Ahmed, Darren F. Seals

**Affiliations:** Department of Biology, Appalachian State University, Boone, North Carolina, United States of America; Thomas Jefferson University, UNITED STATES

## Abstract

The Src substrate Tks5 helps scaffold matrix-remodeling invadopodia in invasive cancer cells. Focus was directed here on how the five SH3 domains of Tks5 impact that activity. Mutations designed to inhibit protein-protein interactions were created in the individual SH3 domains of Tks5, and the constructs were introduced into the LNCaP prostate carcinoma cell line, a model system with intrinsically low Tks5 expression and which our lab had previously showed the dependence of Src-dependent Tks5 phosphorylation on invadopodia development. In LNCaP cells, acute increases in wild-type Tks5 led to increased gelatin matrix degradation. A similar result was observed when Tks5 was mutated in its 4^th^ or 5^th^ SH3 domains. This was in contrast to the 1^st^, 2^nd^, and 3^rd^ SH3 domain mutations of Tks5 where each had a remarkable accentuating effect on gelatin degradation. Conversely, in the invadopodia-competent Src-3T3 model system, mutations in any one of the first three SH3 domains had a dominant negative effect that largely eliminated the presence of invadopodia, inhibited gelatin degradation activity, and redistributed both Src, cortactin, and Tks5 to what are likely endosomal compartments. A hypothesis involving Tks5 conformational states and the regulation of endosomal trafficking is presented as an explanation for these seemingly disparate results.

## Introduction

The acquisition of an invasive phenotype among tumor cells can be a turning point in disease trajectory resulting in poorer cancer patient prognosis and increased mortality. Invasive cells exhibit the concerted ability to move through a tissue environment dense in extracellular matrix proteins, including the basement membrane that defines tissue boundaries. In such cases, invasion may require proteolysis of the matrix to open channels for continued motility. There are cytoskeletal structures that aid in the proteolytic invasion of cancer cells called invadopodia [1, 2]. These structures form through an extensive signaling network long known to be driven by the *SRC* oncogene [3]. Src tyrosine kinase acting through its substrates and associated co-factors leads to focalized actin polymerization, formation of fine protrusions at the cell surface, and matrix-remodeling proteolytic activity. A more thorough understanding of how invadopodia form in cancer cells may present novel opportunities for their neutralization, and thereby a therapy that might limit these devastating aspects of tumor progression in cancer patients.

Over twenty years ago, a novel Src substrate was identified called Tks5/Fish [4]. Like Src, Tks5 is localized to invadopodia and controls their development [5, 6]. Silencing of Tks5 can also diminish the invasive properties of cancer cells resulting in reductions in tumor growth, angiogenesis, and metastasis [6–8]. Tks5 is a scaffolding protein with an amino terminal phox homology (PX) domain, five Src homology 3 (SH3) domains, and several proline-rich motifs [4, 9]. The PX domain accounts for the lipid-binding properties of Tks5 with specificity for the phosphoinositides phosphatidylinositol-3-phosphate (PtdIns(3)P) and PtdIns(3,4)P_2_ [5]. It is the PX domain of Tks5 that is necessary and sufficient for invadopodia localization, and is considered to be a stabilizing event in invadopodia formation [5, 10]. Another requirement for invadopodia formation is the phosphorylation of Tks5 [11, 12]. For example, Src-dependent Tks5 phosphorylation at tyrosine 557 (pY557) confers a binding site for the Src homology 2 (SH2) domain of Nck, and the assembly of a Src-Tks5-Nck signaling pathway that is also instrumental for invadopodia development [11]. Another adaptor protein, Grb2, uses its SH3 domain to bind to one of the proline-rich motifs of Tks5 and acts as an additional recruitment tool for Tks5 during invadopodia assembly [13].

SH3 domains are known for their moderate affinity binding to proline-rich motifs during the assembly of transient protein complexes involved in cell signaling [14]. They are widespread within the human proteome. Pairwise amino acid sequence comparisons indicate 27.5–48% identity across the five SH3 domains of Tks5 [4]. These differences could reasonably account for the differential binding of Tks5 to various proteins and therefore the possible control of different cellular activities, including the scaffolding of invadopodia machinery. For example, binding of the 5^th^ SH3 domain of Tks5 to the metalloproteinase ADAM12 acts in conjunction with Src to increase the shedding of growth factors at invadopodia during hypoxia-induced cancer cell invasion [5, 15]. Another protein, XB130, also associates with the 5^th^ SH3 domain of Tks5. This ultimately leads to the formation of a ternary complex with Src that activates the PI3 kinase signaling pathway and the control of cancer cell proliferation and survival [16]. Some other proteins that have a Tks5 SH3 domain-binding capacity include dynamin (1^st^, 3^rd^, and 5^th^ SH3 domains), N-WASp (all 5 SH3 domains), WIP (3^rd^ and 5^th^ SH3 domains), tubulin (3^rd^ SH3 domain), zyxin (3^rd^ and 5^th^ SH3 domains), nogo-B (5^th^ SH3 domain), F-actin (5^th^ SH3 domain), AFAP-110 (5^th^ SH3 domain), p190RhoGAP (5^th^ SH3 domain), and cortactin (5^th^ SH3 domain) [13, 17]. Each of these proteins has at least some association with actin cytoskeleton regulation and thereby the assembly, dynamics, activity, and/or turnover of invadopodia, and thus puts Tks5 in the center of a number of processes that impact cancer cell invasion.

Notwithstanding a basic understanding of several Tks5 binding partners, there have been no systematic studies to address the impact of Tks5 SH3 domains on invadopodia development. We recently identified a model system for studying the matrix degradation properties of invadopodia using the LNCaP prostate carcinoma cell line [12]. This cell line has very little expression of Src and Tks5 and does not exhibit invadopodia activity on its own, but the transfection of a wild-type Tks5 construct leading to increased Tks5 expression supports gelatin degradation [12]. This ‘assay’ for Tks5-dependent degradation of a gelatin matrix by invadopodia had previously been used to substantiate the importance of Src-dependent Tks5 phosphorylation [12]. Here we use this model system as well as invadopodia-competent Src-transformed fibroblasts to address the function of Tks5 SH3 domains in this process.

## Materials and methods

### Cell culture

The human LNCaP prostate carcinoma cell line, kindly provided by Dr. Doug Lyles (Wake Forest School of Medicine, Winston-Salem NC; [18]), was cultured in RPMI-1640 media formulated with 2mM L-glutamine, 10mM HEPES, and 100μg/ml sodium pyruvate. The murine Src-transformed NIH-3T3 (Src-3T3) cell line, kindly provided by Dr. Sara Courtneidge (Oregon Health and Sciences University, Portland OR; [4]) was cultured in DMEM media formulated with 2mM L-glutamine and 110μg/ml sodium pyruvate. Both media formulations were supplemented with 10% fetal calf serum (Sigma) and 1% penicillin/streptomycin (final concentrations). Both cell lines were maintained in a climate-controlled incubator at 37°C and 5% CO_2_.

### Constructs

A murine Tks5 cDNA had previously been introduced into the pSGT mammalian expression vector with an in-frame myc epitope tag at the carboxy terminus. To study the function of Tks5 SH3 domains, point mutations were created in each of the individual SH3 domains of Tks5 by site-directed mutagenesis (QuickChange II, Stratagene) such that the first in a conserved pair of tryptophans was converted to an alanine. These five constructs were given the names m1 (W188A), m2 (W260A), m3 (W441A), m4 (W827A), and m5 (W1056A). [Note that the position of the mutations is based on the form of murine Tks5 containing the PX domain, but lacking both alternative splice insertions [4, 9].] We later noted that the expression of Tks5 in LNCaP cells electroporated with the wild-type Tks5 construct was much higher when driven by the CMV promoter of pcDNA3 than from the SV40 promoter of pSGT. We therefore moved the regions surrounding the mutations in the m1-m5 constructs in pSGT to the corresponding regions of the wild-type and untagged Tks5 construct in the pcDNA3 vector so that all Tks5 mutations could be expressed to the more robust levels necessary to support gelatin degradation in LNCaP cells. This was accomplished by exchanging BlpI-BstEII fragments for the m1-m3 constructs and exchanging a BstEII-BsrGI fragment for the m4 construct. The m5 construct had been created before [5]. All Tks5 mutant constructs were verified by DNA sequencing (GeneWiz). The GFP-tagged, tandem FYVE domains of Hrs that binds to PtdIns(3)P has been described previously and was a generous gift of Dr. Ed Skolnik (Skirball Institute of Biomolecular Medicine, New York NY) [19].

### Transfections

Each Tks5 construct was individually introduced into LNCaP cells using a proprietary Nucleofection^TM^ technique (Lonza) as previously described [12]. Briefly, 2 x 10^6^ LNCaP cells collected from a culture dish at ~75% confluency were mixed with 3–10μg of plasmid DNA and solutions from Nuclefector Kit R to a final volume of 100μl and then electroporated using program T-009. After electroporation, the cells were allowed to recover for 15–30 minutes in 500μl of pre-warmed culture media before being plated for further experimentation as described below. For the Src-3T3 cells, all constructs were introduced using Lipofectamine^TM^ 3000 (ThermoFisher). In this case, two aliquots of Opti-MEM® reduced serum media (ThermoFisher) were separately mixed with 4μl of Lipofectamine and with 7μg DNA/10μl Lipofectamine, each in a final volume of 125μl, before mixing them together for a 10-minute incubation at room temperature. The DNA-lipid complexes were then added to a 6cm dish of 75% confluent Src-3T3 cells and the cells cultured for 24 to 48 hours. Cells transfected with an empty pcDNA3 or pSGT vector served as negative controls in these experiments.

### Immunoblotting

General conditions for cell lysis, protein quantitation, SDS-PAGE, and immunoblotting have been described previously with certain modifications [20]. Cells attached to culture dishes were first washed with 0.6 culture volumes of ice-cold phosphate-buffered saline (PBS) containing 1mM sodium orthovanadate before mixing with 0.05 culture volumes of NP40 lysis buffer composed of 1% NP40, 20mM HEPES (pH 7.0), 110mM sodium chloride, 40mM sodium fluoride, 1mM sodium orthovanadate, and a cocktail of protease inhibitors that included a final concentration of 10μg/ml aprotinin/PBS, 10μg/ml leupeptin/PBS, 10μg/ml pepstatin/DMSO, 1mM benzamidine/PBS, and 1mM PMSF/DMSO. The cells were scraped in this buffer and collected into microfuge tubes for a 10-minute incubation on ice followed by clearance at 10,000x*g* for 10 minutes at 4°C. The supernatant was then assayed for protein using a detergent-compatible assay kit (Bio-Rad) against known standard concentrations of bovine serum albumin (BSA). Whole cell lysate protein concentrations generally varied from 1.5–3 μg/μl. Protein samples were mixed with SDS-PAGE loading dye to a final concentration of 1 μg/μl, and 35 to 55μg of protein were loaded on a denaturing 7.5% polyacrylamide gel and separated at 150-200V for ~1 hour. Proteins were then electrophoretically transferred to a 0.45μm nitrocellulose membrane under semi-dry conditions for immunoblotting purposes (Bio-Rad). After incubation in a 5% milk/0.5% BSA/PBS-0.1% Tween blocking solution, the membrane was incubated overnight at 4°C with a primary polyclonal antibody specific to Tks5 (1:1000, sc-30122, Santa Cruz) or GAPDH (1:1000, sc-25778, Santa Cruz) diluted in 10% blocking solution. This was followed by incubation in a species-specific peroxidase-conjugated secondary antibody (1:2500, NA9340V and NA931V, GE Healthcare) diluted in 10% blocking solution for 30 minutes at room temperature. Three, 5-minute washes in PBS-0.1% Tween were done after each antibody application. Proteins were visualized using SuperSignal^TM^ West Dura extended duration substrate (ThermoFisher) and a ChemiDoc imaging system (Bio-Rad) according to manufacturer instructions. Image J was used for the densitometric analysis of Tks5 expression, reported as a ratio to that of GAPDH and normalized to wild-type Tks5 protein levels.

### *In situ* zymography

Transfected LNCaP cells (3.2 x 10^5^) were grown under standard growth conditions for 48 hours in 12-well plates where each well contained a glass coverslip coated sequentially with 50μg/ml poly-L-lysine and 111μg/ml Oregon Green 488-labeled gelatin [21]. For the transfected Src-3T3 cells, cells were re-plated after a 24- to 48- hour post-transfection recovery period into 12-well plates containing the same gelatin-coated coverslips as the LNCaP cells, but were only cultured for 4 hours. All cells on the coverslips were subsequently fixed in 0.3% formaldehyde/PBS and permeabilized in 0.4% Triton-X-100/PBS, each for 10 minutes at room temperature. LNCaP cells were stained with AlexaFluor 594-conjugated phalloidin (1:200, Invitrogen) for 1 hour in 5% donkey serum/PBS. Src-3T3 cells were stained with a primary antibody to the myc epitope tag of Tks5 (1:1000, Millipore, clone 4A6) for 3 hours and then with AlexaFluor 594-conjugated mouse secondary antibody (1:2000, GE Healthcare) for 1 hour, all in 5% donkey serum/PBS. Three, 5-minute washes in PBS were done after phalloidin and/or antibody applications. Coverslips were mounted on glass slides containing a single droplet of ProLong^TM^ Gold Antifade Mountant with DAPI (Life Technologies). Ten random images of each experimental condition (~30 cells/image) were taken on an Olympus BX51 microscope equipped with a Retiga EXi Fast 1394 camera (Q-Imaging). Image processing was conducted with Q-Capture 64 Suite, Adobe Photoshop CS6, and/or Adobe Illustrator CS6 software. The area of gelatin degradation was quantified using ImageJ 1.49 software across multiple images in LNCaP cells or for each transfected Src3T3 cell.

### Fluorescence microscopy

Transfected LNCaP (2.7 x 10^5^) or Src-3T3 cells were grown under standard growth conditions for 48 hours in 6-well plates (LNCaP cells) or 6cm dishes (Src-3T3 cells) containing uncoated glass coverslips. All cells on the coverslips were fixed in 0.3% formaldehyde/PBS and permeabilized in 0.4% Triton-X-100/PBS as described above. In LNCaP cells, the localization of Tks5 and F-actin were based on staining with a primary antibody to Tks5 (1:1000, Santa Cruz, #sc-30122) or an antibody specific to the myc epitope tag (1:1000, Millipore, clone 4A6) for 3 hours and then with AlexaFluor 488-conjugated mouse secondary antibody (1:2000, GE Healthcare) and AlexaFluor 594-conjugated phalloidin (1:200, Invitrogen) for 1 hour, all in 5% donkey serum/PBS. In Src-3T3 cells, when studying the co-localization of Tks5 with other proteins, the coverslips were stained with an antibody specific to the myc epitope tag (1:1000, Millipore, clone 4A6) and with primary antibodies to cortactin (1:1000, Santa Cruz, #05–180), Src (1:1000, Cell Signaling, #2108) or EEA1 (1:1000, BD Transduction Labs, #610456) for 3 hours, and then with AlexaFluor 488-conjugated rabbit secondary antibody (1:2000, GE Healthcare) and AlexaFluor 594-conjugated mouse secondary antibody (1:2000, GE Healthcare) for 1 hour, all in 5% donkey serum/PBS. The fluorescence emitted from the GFP-tagged, tandem FYVE domains of Hrs was analyzed directly. Coverslips were mounted and imaged as described above. In selected images, pixel intensities relative to background were quantified for each marker protein along a transect using the Plot Profile macro available in ImageJ 1.49 software with the data presented as stacked histograms.

## Results

### Effect of Tks5 SH3 domain mutations on invadopodia formation and matrix degradation activity in LNCaP cells

**Fig 1:**
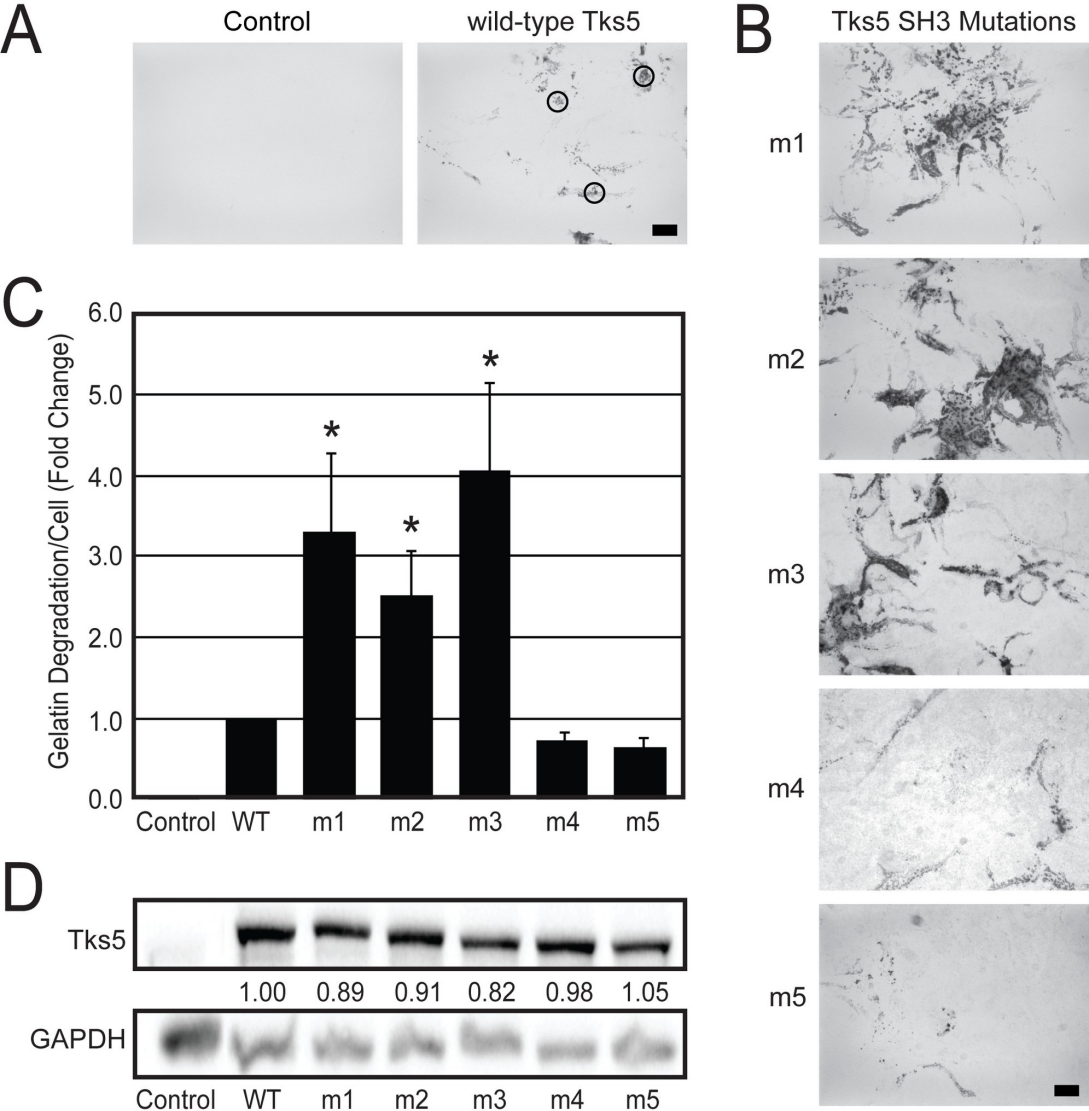
Tks5 SH3 domain mutations differentially alter gelatin matrix degradation in LNCaP cells. LNCaP cells were transiently transfected with the indicated wild-type and mutant (m1-m5) SH3 domain Tks5 constructs and analyzed for gelatin matrix degradation by *in situ* zymography. Control LNCaP cells were transfected with the pcDNA3 vector alone. (A,B) Representative mages show the sites where gelatin degradation (black regions within the green fluorescent gelatin monolayer; *e*.*g*. black circles) occurred over 48 hours of culture post-transfection. Scale bars (right and bottom panels) = 20μm. (C) The areas of gelatin degradation were normalized per cell from 10 random images for each experimental condition and expressed as the fold change relative to the degradation observed in cells expressing wild-type Tks5 (n = 3). Statistical comparisons were done using a Student’s *t*-test (*P < 0.05). (D) Representative immunoblot analysis of Tks5 and GAPDH (loading control) among whole LNCaP cell lysates after 48 hours of culture post-transfection. The numerical values are the average densitometric measurements of Tks5:GAPDH ratios normalized to wild-type Tks5 expression (n = 3).

Previously we had used the LNCaP prostate carcinoma cell line to demonstrate that the Src substrate and adaptor protein Tks5 could sufficiently induce gelatin degradation activity and thus increase the invasive behavior of the cell line [12]. Given the success of that assay we considered the utility of this model system in determining the relative contribution of the SH3 domains of Tks5 to those processes. To that end, point mutations were generated in murine Tks5 constructs such that a conserved tryptophan residue known to convey SH3 domain binding to proline-rich motifs was changed to an alanine. This mutation was individually created in each of the five SH3 domains of Tks5, after which all five constructs (named m1, m2, m3, m4, and m5) were sub-cloned into the mammalian expression vector pcDNA3, verified by DNA sequencing, and introduced one-by-one into LNCaP cells by electroporation. To gain an initial indication of invadopodia development in this cell line, we first used a highly sensitive *in situ* zymography assay that has long been used to measure invadopodia-associated matrix degradation activity. LNCaP cells transiently transfected with either wild-type or SH3 domain mutant Tks5 constructs were cultured on a thin layer of fluorescently-labeled gelatin for 48 hours where evidence of invadopodia activity was indicated by areas of clearance in the fluorescent gelatin monolayers. First, we showed the requirement of a wild-type Tks5 construct for gelatin degradation, similar to that observed previously (Fig 1A, *e*.*g*. black circles) [12]. We had initially hypothesized that mutations in the SH3 domains would disrupt this function of Tks5 and disable its ability to stimulate gelatin degradation in LNCaP cells, but this was not observed. Two of the mutant Tks5 constructs, m4 (mutation in the 4^th^ SH3 domain) and m5 (mutation in the 5^th^ SH3 domain), seemed to induce gelatinolytic activity in the transfected cells like the wild-type construct did (Fig 1B). The others, however, including the m1 (mutation in the 1^st^ SH3 domain), m2 (mutation in the 2^nd^ SH3 domain), and m3 (mutation in the 3^rd^ SH3 domain) constructs, stimulated gelatin degradation to even higher levels. Quantitative analysis of gelatin degradation confirmed these initial observations where the m1, m2, and m3 mutant Tks5 constructs exhibited an induction in gelatin degradation that varied from 2.5- to 4.1-fold over that of wild-type Tks5, and in all cases was verified to be statistically significant (Fig 1C). Importantly, these accentuated changes in gelatin degradation by the m1, m2, and m3 Tks5 constructs were not simply attributed to differences in Tks5 expression (Fig 1D). As in our previous study, little endogenous Tks5 protein was present in control, empty vector-transfected LNCaP cells, while the cells transfected with the wild-type and SH3 domain mutant Tks5 constructs exhibited similarly high expression levels 48 hours after electroporation and across multiple experiments (Fig 1D) [12]. Thus, in the context of the LNCaP model system, there are differential contributions for each Tks5 SH3 domain on the gelatin degradation component of invadopodia development with the 4^th^ and 5^th^ SH3 domains having no significant effect and the 1^st^, 2^nd^, and 3^rd^ SH3 domains exhibiting a previously unappreciated inhibitory role.

**Fig 2:**
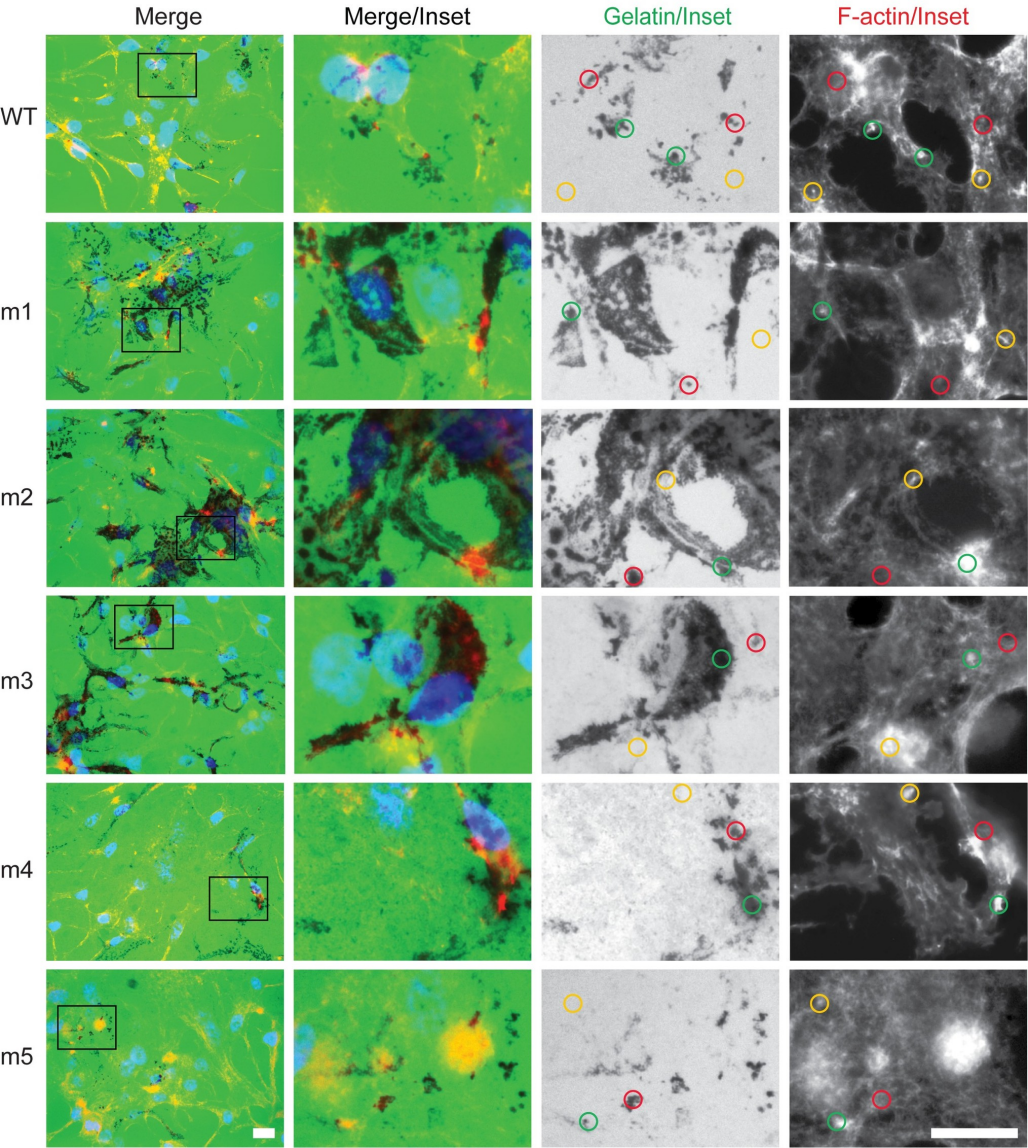
Gelatin degradation patterns are largely diffuse in LNCaP cells following acute changes in Tks5 protein levels. LNCaP cells were transiently transfected with the indicated wild-type and mutant (m1-m5) SH3 domain Tks5 constructs and analyzed for gelatin matrix degradation by *in situ* zymography. Merged images from Fig 1 show the presence of nuclei (DAPI, blue), F-actin (phalloidin, red), and gelatin degradation (black regions within the green fluorescent gelatin monolayer). The black boxes in the left-most panels indicate regions of image enlargement depicted in the inset panels at right. Green circles indicate puncta with co-incident F-actin staining and gelatin degradation; yellow circles indicate puncta with F-actin staining alone; and red circles indicate sites with gelatin degradation alone. Scale bars (bottom left and bottom right panels) = 20μm.

There may be a number of ways to explain the accentuated gelatin matrix degradation activity of LNCaP cells transfected with the m1, m2, or m3 (collectively, m1-m3) Tks5 constructs. In a fairly straightforward approach, we next tested whether the m1-m3 mutant Tks5 constructs were better able to generate invadopodia or to reach those sites in the cell where they form. We noticed that LNCaP cells transiently transfected with wild-type and mutant Tks5 constructs sometimes displayed punctate filamentous actin (F-actin) structures, and as a possible indicator of invadopodia those sites were sometimes co-incident with sites of gelatin degradation (Fig 2, green circles). However, after 48 hours of culture it was also possible to locate sites of F-actin puncta that were not associated with gelatin degradation (Fig 2, yellow circles), and to locate sites of gelatin degradation that were not associated with F-actin puncta (Fig 2, red circles). More generally, the gelatin degradation patterns tended to be diffuse in the Tks5-transfected LNCaP cells. We also note that we did not know which cells were transfected in this assay as they were not individually marked or stained in any way.

**Fig 3:**
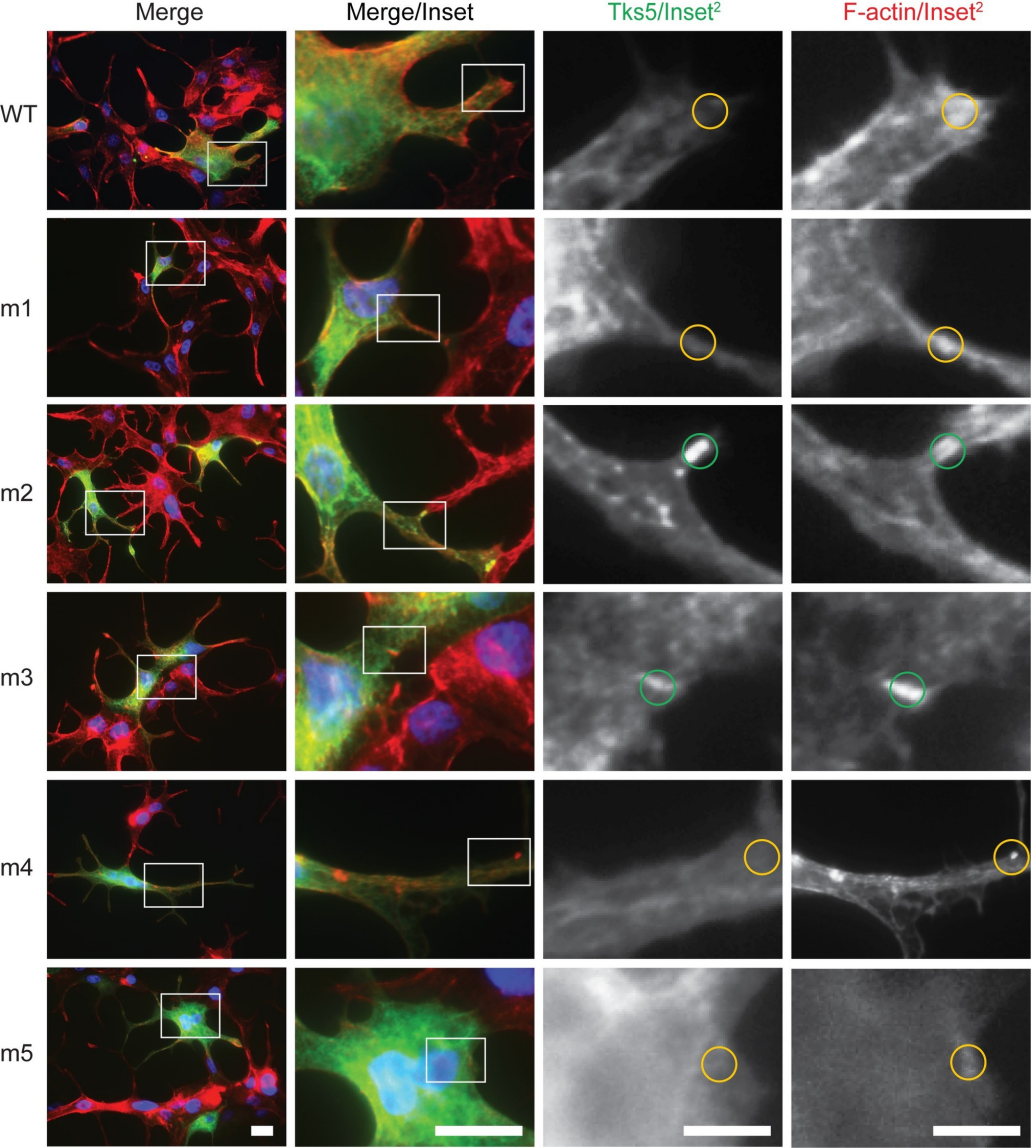
LNCaP cells do not readily form invadopodia-like punctate structures following acute changes in Tks5 protein levels. LNCaP cells were transiently transfected with the indicated wild-type and mutant (m1-m5) SH3 domain Tks5 constructs and analyzed for its localization (Tks5 antibody, green) alongside F-actin (phalloidin, red) by fluorescence microscopy. Nuclei (DAPI, blue). The white boxes in the left panels indicate regions of image enlargement (Merge/Inset) depicted in the left/middle inset panels (scale bars = 20μm). The white boxes in the left/middle inset panels indicate further regions of image enlargement (Tks5/Inset^2^ and F-actin/Inset^2^) in the right/middle and right inset panels (scale bars = 80μm). Green and yellow circles represent the rare conditions of puncta with co-incident F-actin and Tks5 staining versus F-actin staining alone, respectively.

To address this issue, wild-type Tks5 and the constructs harboring the Tks5 SH3 domain mutations were transiently transfected into LNCaP cells and monitored for invadopodia based on F-actin/Tks5 co-staining [12]. Again, there were cases where the co-incident staining of F-actin and Tks5 occurred in punctate structures of transfected cells, but this was rare in this cell line (Fig 3, green circles). More commonly, the staining of F-actin and Tks5 was similar to the pattern of gelatin degradation in the transfected LNCaP cells; *i*.*e*. mostly diffuse in nature. Thus, while this LNCaP model system described the differential impact of Tks5 SH3 domains on gelatin matrix degradation activity, it was unable to render decisive conclusions on the nature of invadopodia structures themselves.

### Effect of Tks5 SH3 domain mutations on invadopodia formation and matrix degradation activity in Src3T3 cells

**Fig 4:**
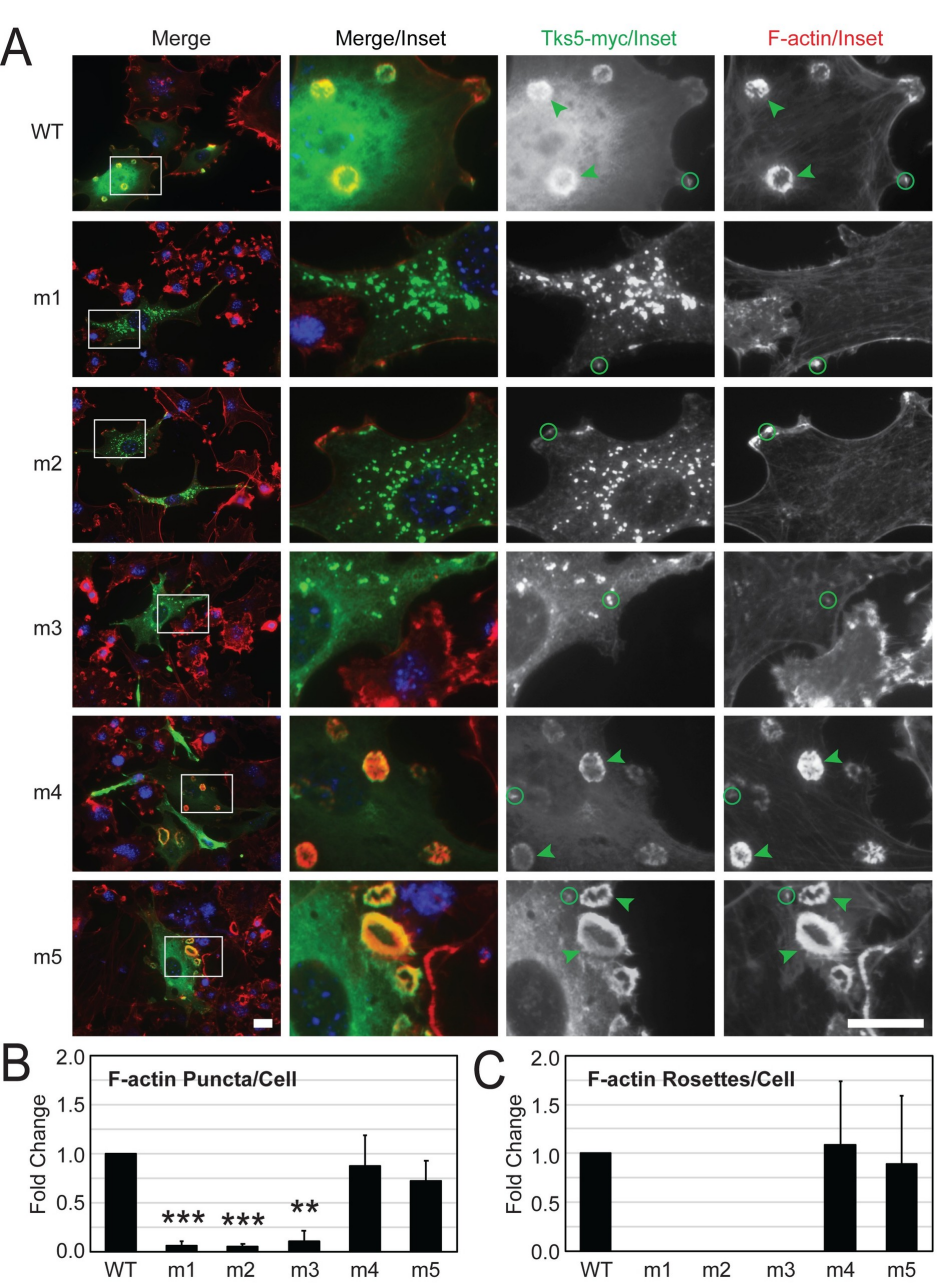
Tks5 SH3 domain mutations differentially co-localize with invadopodia in Src-3T3 cells. (A) Src-3T3 cells were transiently transfected with the indicated wild-type (WT) and mutant (m1-m5) SH3 domain Tks5-myc constructs and analyzed for its localization (myc antibody, green) alongside F-actin (phalloidin, red) by fluorescence microscopy. Nuclei (DAPI, blue). The white boxes in the left-most panels indicate regions of image enlargement depicted in the inset panels at right. Representative punctate- and rosette-shaped invadopodia based on co-incident F-actin/Tks5-myc staining are indicated with green circles and arrowheads, respectively. Scale bars (bottom left and bottom right panels) = 20μm. (B,C) Punctate- and rosette-shaped F-actin structures as representations of invadopodia were counted among transfected cells in 5 random images for each experimental condition and expressed as the fold change relative to the cells expressing wild-type Tks5 (n = 3). Statistical comparisons were done using a Student’s *t*-test (**P < 0.01, ***P < 0.001).

Since LNCaP cells do not readily form visible invadopodia upon acute changes in Tks5 expression, we decided to examine the localization of the wild-type and mutant SH3 domain Tks5 constructs in a more robust, invadopodia-competent model system represented by Src-3T3 cells [6]. Src-3T3 is an NIH-3T3-derived fibroblast cell line that has been stably modified for expression of a constitutively active form of chicken Src (Y527F mutation). In addition to their robust Src tyrosine kinase activity, these cells have naturally high endogenous Tks5 protein levels and form well-developed invadopodia structures that occasionally coalesce into ring-shaped rosettes (Fig 4A, arrowheads) [6]. To facilitate visualization of ectopic Tks5 against the substantial background levels of endogenous protein, we used our original wild-type and SH3 domain mutant Tks5 constructs in the pSGT vector that contained in-frame myc epitope tags. From past studies we know that Tks5-myc localizes to invadopodia rosettes and that its binding is based on the phosphoinositide-binding properties of its PX domain [5]. Since the m1-m3 mutant Tks5 constructs accentuated matrix-degrading invadopodia activity in LNCaP cells we hypothesized localization to the invadopodia of Src-3T3 cells. However, when Src-3T3 cells were transfected with the wild-type and SH3 domain mutant Tks5-myc constructs and assayed for their localization to invadopodia by immunofluorescence microscopy, the myc epitope antibody used to detect ectopic Tks5 only revealed strong localization to invadopodia rosettes with wild-type Tks5 and with the m4 and m5 mutant Tks5 constructs (Fig 4A, arrowheads). The m1-m3 mutant Tks5 constructs, in contrast, largely failed to localize to invadopodia. Indeed, both punctate- and rosette-shaped invadopodia structures, as revealed by the staining of F-actin, were significantly reduced or completely absent in these m1-m3 transfected cells (Fig 4B and 4C). Interestingly, this reduction in invadopodia was also associated with a unique Tks5 localization pattern where the m1-m3 mutant Tks5 constructs were condensed at discrete sites in the cytoplasm and in the peri-nuclear region where little F-actin was present (Fig 4A).

**Fig 5:**
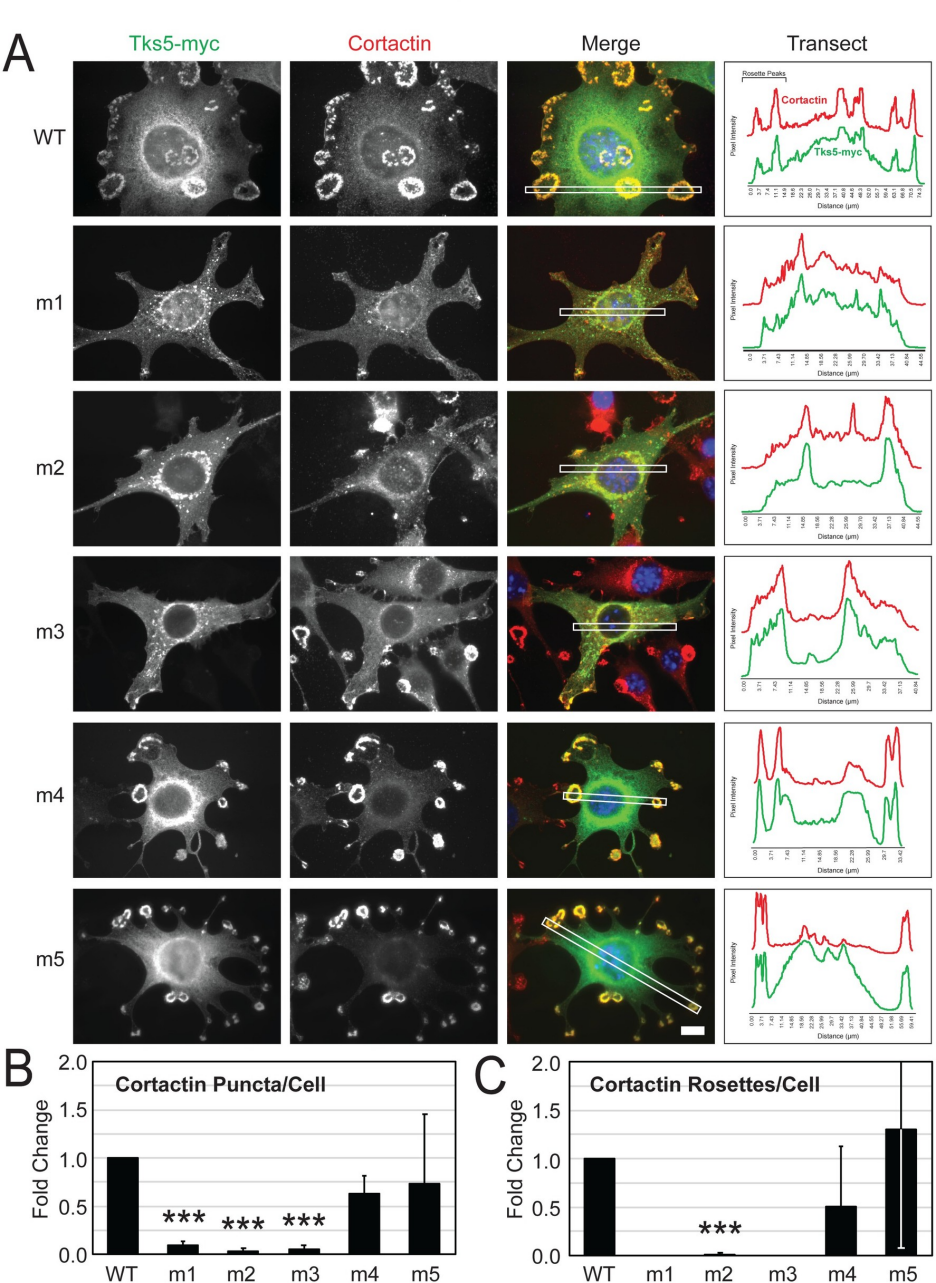
Cortactin is mis-localized in Src-3T3 cells transfected with Tks5 harboring mutations in the first, second, or third SH3 domains. Src-3T3 cells were transiently transfected with the indicated wild-type (WT) and mutant (m1-m5) SH3 domain Tks5-myc constructs and analyzed for its localization (myc antibody, green) alongside cortactin (red) by immunofluorescence microscopy. Nuclei (DAPI, blue). Scale bar (bottom right panel) = 10μm. The panels at right show the fluorescence intensity of Tks5-myc and cortactin relative to background along a transect (white box in merged image). (B,C) Punctate- and rosette-shaped cortactin structures as representations of invadopodia were counted among transfected cells in 5 random images for each experimental condition and expressed as the fold change relative to the cells expressing wild-type Tks5 (n = 3). Statistical comparisons were done using a Student’s *t*-test (***P < 0.001).

To follow-up on this surprising impact of the m1-m3 mutant SH3 domain constructs on invadopodia in Src3T3 cells, we also investigated cortactin as it too localizes to invadopodia in this model system. This localization pattern was demonstrated when wild-type Tks5 was transfected into these cells where a transect through the image demonstrates paired peaks of Tks5-myc and cortactin fluorescence for each invadopodia rosette (Fig 5A). Similar results were observed after transfection of the m4 and m5 mutant Tks5 constructs (Fig 5A). In contrast, when Src-3T3 cells were transfected with the m1-m3 mutant Tks5 constructs, both punctate- and rosette-shaped invadopodia were significantly depleted (Fig 5B and 5C), and both Tks5-myc and cortactin were redistributed in the cells (Fig 5A). Some cortactin was found throughout the cytoplasm, some was localized at the cell cortex, and there was significant enrichment around the nucleus along-side Tks5-myc (Fig 5A). Altogether, the transect data generally indicate Tks5-myc/cortactin co-localization in Src-3T3 cells, but this occurred at invadopodia in the wild-type-, m4-, and m5-transfected cells and in the peri-nuclear region of m1-m3-transfected cells.

**Fig 6:**
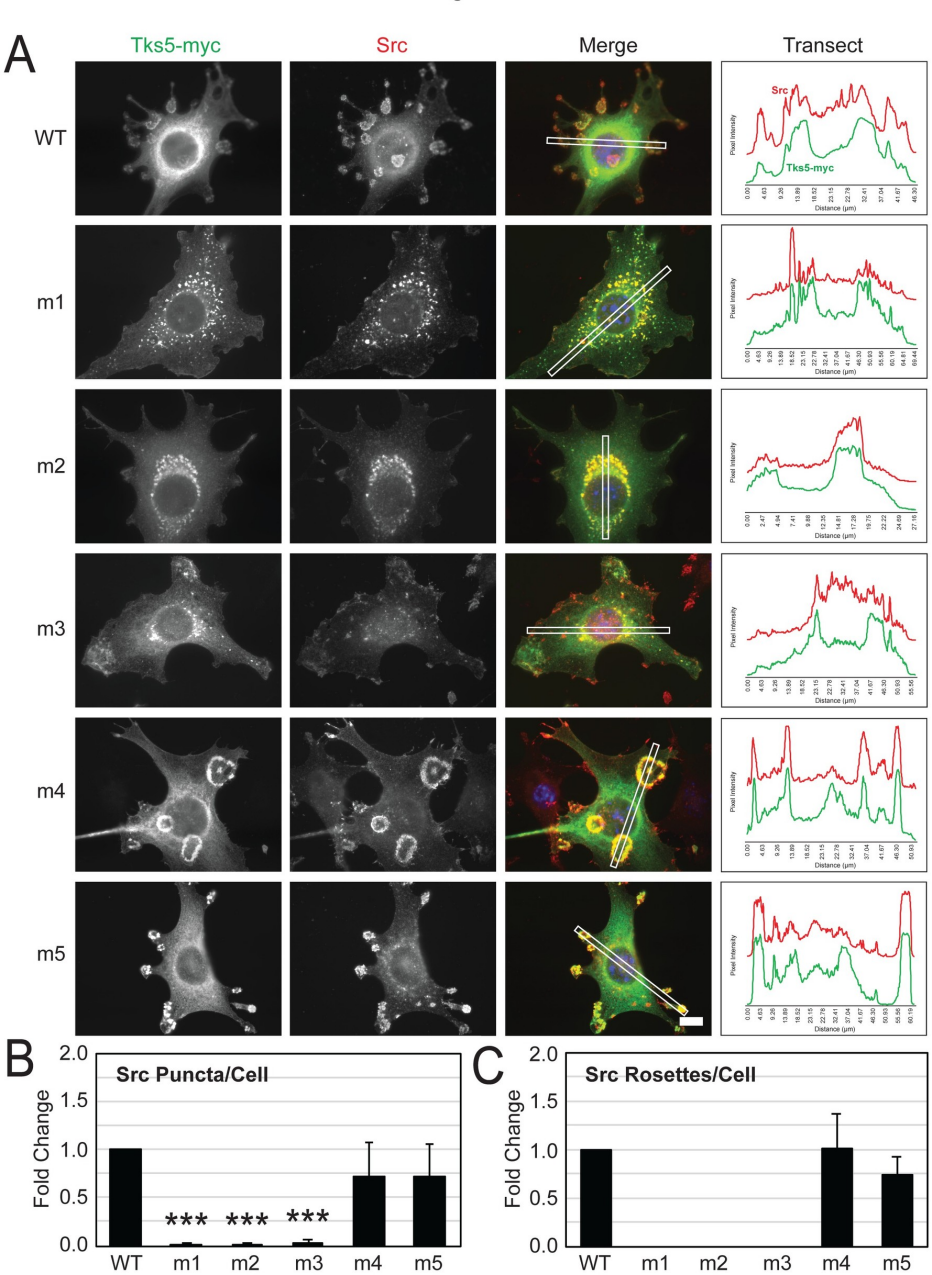
Activated Src is mis-localized in Src-3T3 cells transfected with Tks5 harboring mutations in the first, second, or third SH3 domains. Src-3T3 cells were transiently transfected with the indicated wild-type (WT) and mutant (m1-m5) SH3 domain Tks5-myc constructs and analyzed for its localization (myc antibody, green) alongside activated Src (Src-pY418 antibody, red) by immunofluorescence microscopy. Nuclei (DAPI, blue). Scale bar (bottom right panel) = 10μm. The panels at right show the fluorescence intensity of Tks5-myc and Src relative to background along a transect (white box in merged image). (B,C) Punctate- and rosette-shaped Src structures as representations of invadopodia were counted among transfected cells in 5 random images for each experimental condition and expressed as the fold change relative to the cells expressing wild-type Tks5 (n = 3). Statistical comparisons were done using a Student’s *t*-test (***P < 0.001).

Src tyrosine kinase catalytic activity drives invadopodia rosette development in Src-3T3 cells, acting in conjunction with its many substrates and co-factors, including Tks5 and cortactin. Src is also localized to invadopodia rosettes. We therefore examined the localization of Src when Src-3T3 cells were transfected with the wild-type and mutant Tks5 constructs. The results were similar to that of cortactin. That is, Src co-localized with Tks5-myc at the invadopodia of wild-type-, m4-, and m5-transfected Src-3T3 cells, but both proteins redistributed to the peri-nuclear region of m1-m3-tranfected Src-3T3 cells when invadopodia were largely depleted (Fig 6).

**Fig 7:**
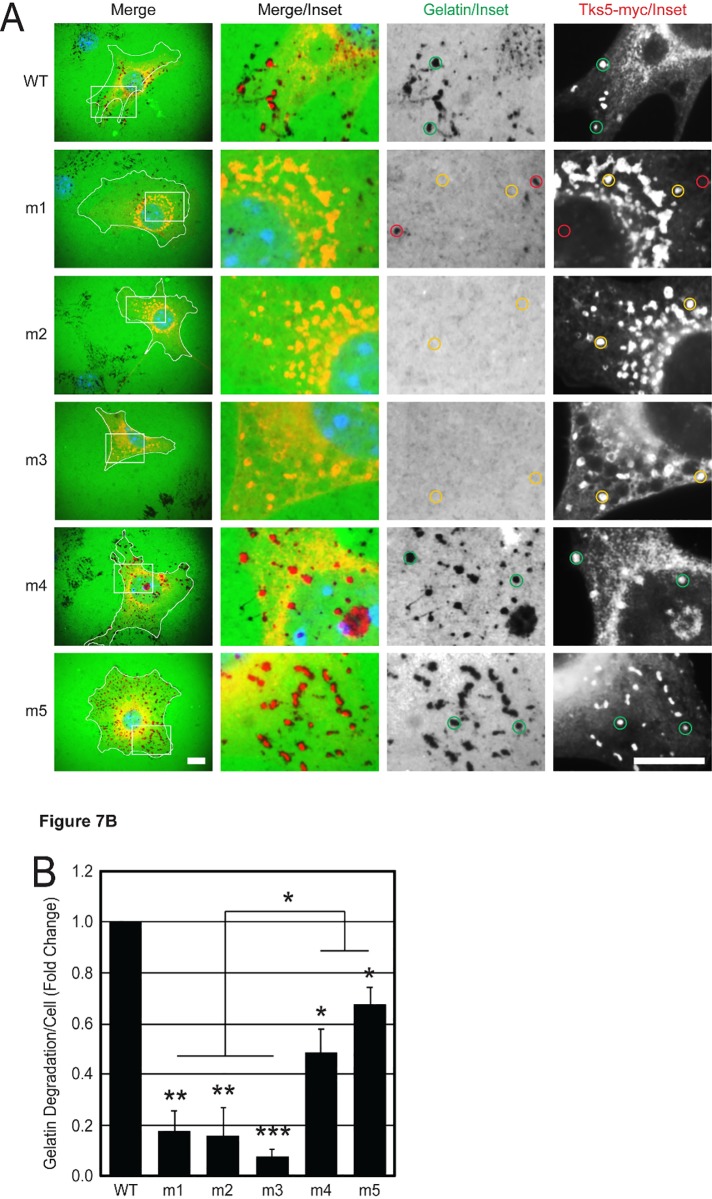
Tks5 SH3 domain mutations differentially co-localize with invadopodia-associated gelatin matrix degradation in Src-3T3 cells. (A) Src-3T3 cells were transiently transfected with the indicated wild-type (WT) and mutant (m1-m5) SH3 domain Tks5-myc constructs and analyzed for invadopodia-associated gelatin matrix degradation (dark spots in the green gelatin monolayer) by *in situ* zymography in individually transfected cells (see cell demarcations in white). The localization of ectopic Tks5 (red) was based on an antibody to the myc epitope tag. Nuclei (DAPI, blue). The white boxes in the left-most panels indicate regions of image enlargement depicted in the inset panels at right. Green circles indicate puncta with co-incident Tks5-myc staining and gelatin degradation; yellow circles indicate puncta with Tks5-myc staining alone; and red circles indicate sites with gelatin degradation alone. Scale bars (bottom left and bottom right panels) = 20μm. (B) The areas of gelatin degradation occupied by transfected cells were normalized per cell from 5 random images for each experimental condition and expressed as the fold change relative to the cells expressing wild-type Tks5 (n = 3). Statistical comparisons were done using a Student’s *t*-test (*P < 0.05, **P < 0.01, ***P < 0.001).

While the Src-3T3 cells transfected with the m1-m3 mutant Tks5 constructs lacked prominent invadopodia structures, there was still retention of Tks5-Src co-localization, and we wondered whether these new cellular regions had gelatinolytic activity. Src-3T3 cells exhibit considerable matrix-degrading invadopodia activity, so in order to discern the activity in individually transfected cells, we transiently transfected Src-3T3 cells for 48 hours with wild-type and mutant Tks5 constructs, then passaged the cells onto gelatin-coated coverslips before fixing them 4 hours later. At this point, the cells have just become adherent and are beginning to form punctate invadopodia structures in which invadopodia markers and gelatin degradation can be co-localized. This was exactly what was observed when wild-type Tks5 was introduced into Src-3T3 cells where among some peri-nuclear staining there was distinct, co-incident, and focalized Tks5 staining (via the myc epitope tag) aligning with the holes formed by gelatin degradation (Fig 7A, green circles). The same was also true of Src-3T3 cells transfected with the m4 and m5 Tks5 SH3 domain mutant constructs. This focalized pattern of degradation in invadopodia-competent Src-3T3 cells was quite different from the diffuse pattern seen in LNCaP cells overexpressing Tks5 (compare Figs 1A to 7A). It was also different from what was observed with Src-3T3 cells transfected with the m1-m3 Tks5 mutant constructs. In this case, Tks5-myc exhibited a punctate distribution pattern, but it was nearly exclusively redistributed to the peri-nuclear region of the cells as observed before (Figs 4A, 5, 6 and 7A). Moreover, these locales did not exhibit any degradation of the gelatin matrix (Fig 7A, yellow circles). Indeed, m1-m3-transfected cells exhibited 5-fold decreases in gelatin matrix degradation relative to wild-type transfected cells (Fig 7B). They also degraded significantly less gelatin than the m4 or m5-transfected cells. So not only do the Src-3T3 cells appear to lose their normal ability to make invadopodia structures following transfection of the m1, m2, and m3 mutant Tks5 constructs, much of their matrix degradation capabilities have been compromised as well.

Taken altogether, the m1-m3 mutant Tks5 constructs have opposing effects. In the LNCaP model system, matrix-remodeling invadopodia activity is accentuated by these Tks5 SH3 domain mutations whereas in the invadopodia-competent Src-3T3 model system, these constructs act as dominant-negative inhibitors of invadopodia development.

### Mutations in its first three SH3 domains redistribute Tks5 to endosomes

**Fig 8:**
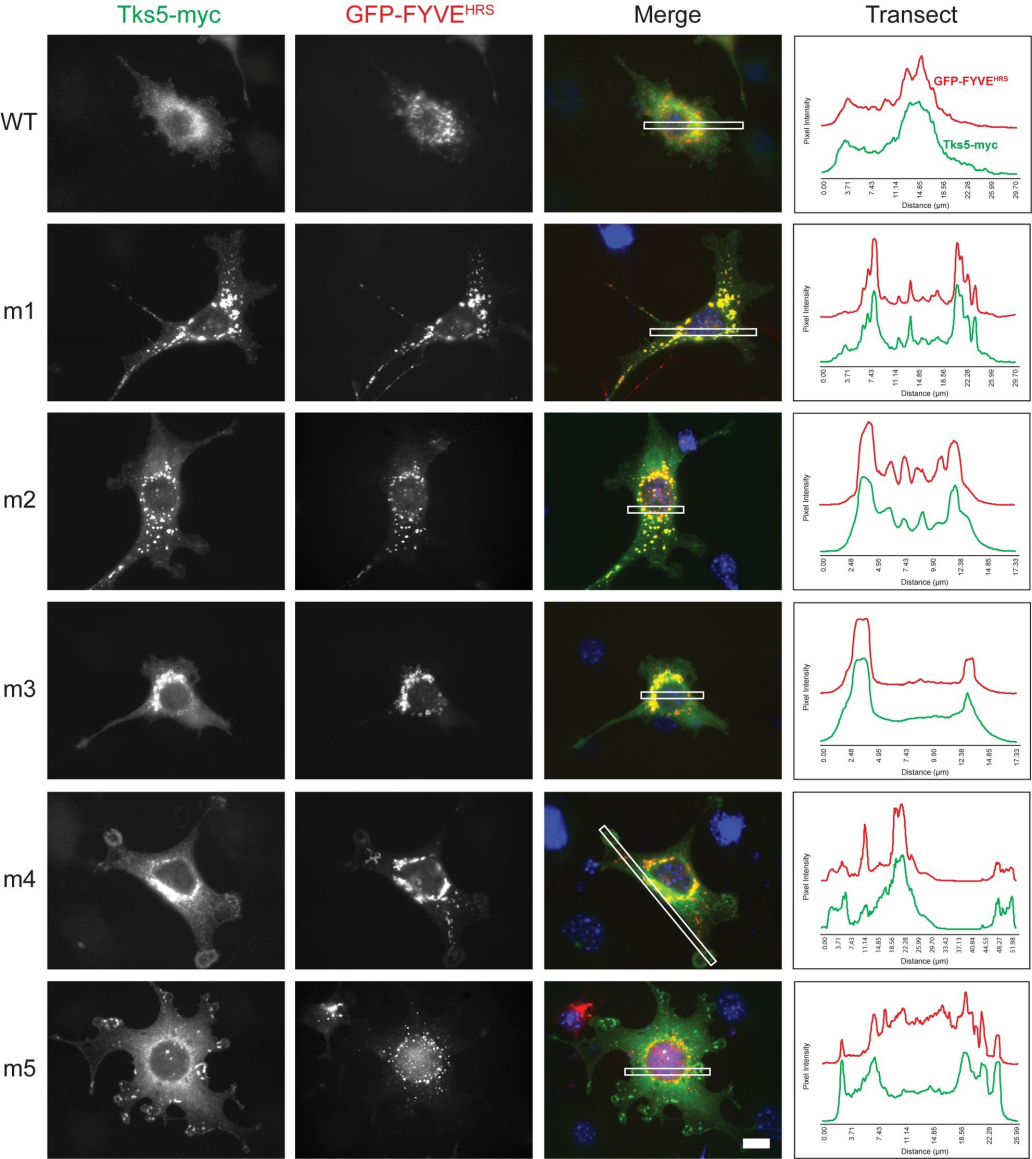
Co-localization of Tks5 harboring mutations in the first, second, or third SH3 domains with the FYVE domain of Hrs. Src-3T3 cells were transiently co-transfected with the indicated wild-type (WT) and mutant (m1-m5) SH3 domain Tks5-myc constructs and with the tandem FYVE domain of the endosomal marker protein Hrs tagged with GFP. The localization of ectopic Tks5 was based on an antibody to the myc epitope tag (green). The localization of FYVE^HRS^ was based on direct detection of GFP fluorescence (pseudocolored red). Nuclei (DAPI, blue). Scale bar (bottom right panel) = 10μm. The panels at right show the fluorescence intensity of Tks5-myc and GFP-FYVE^HRS^ relative to background along a transect (white box in merged image).

We noticed that the localization pattern of the m1-m3 mutant Tks5 constructs in the Src3T3 cells shared some similarity to the previously published localization pattern of the GFP-tagged PX domain of Tks5 in non-transformed NIH-3T3 cells [5]. This localization pattern in the NIH-3T3 cells was hypothesized to be a phosphoinositide-rich endosomal compartment based on a similar localization pattern as the GFP-tagged FYVE domain of the endosomal marker protein Hrs [5]. Presumably the PX domain of Tks5 was bound to similar phosphoinositides as the FYVE domain of Hrs on the endosomal membrane. To examine whether the punctate structures in Src-3T3 cells transfected with the m1-m3 mutant Tks5 constructs might also be endosomes, we examined the localization pattern of the GFP-tagged FYVE domain in these cells. First, all transfected cells showed positive GFP fluorescence in the peri-nuclear region suggesting prominent endosomal compartments at this cellular location. Importantly though, co-incident patterns of fluorescence as quantified by the transect data indicate that both the GFP-FYVE domain of Hrs and the m1-m3 mutant Tks5 constructs were co-localized to the same region in Src-3T3 cells (Fig 8). Some peri-nuclear co-localization could also be seen in the wild-type-, m4-, and m5-transfected cells, but in these cases Tks5-myc staining of invadopodia could still be seen as well (Fig 8).

**Fig 9:**
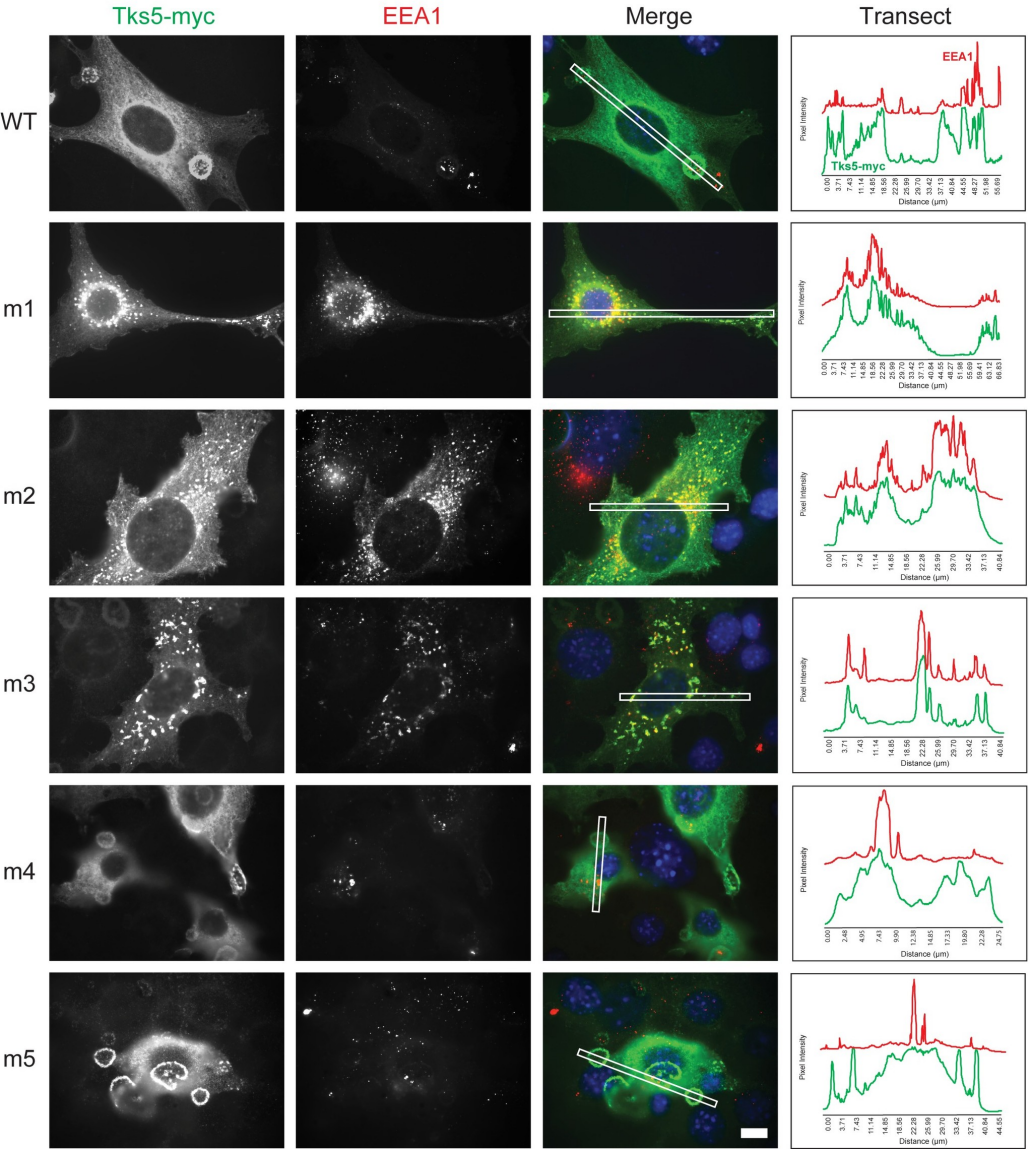
Co-localization of Tks5 harboring mutations in the first, second, or third SH3 domains with the endosomal marker protein EEA1. Src-3T3 cells were transiently transfected with the indicated wild-type (WT) and mutant (m1-m5) SH3 domain Tks5-myc constructs and analyzed for its localization (myc antibody, green) alongside EEA1 (red) by immunofluorescence microscopy. Nuclei (DAPI, blue). Scale bar (bottom right panel) = 10μm. The panels at right show the fluorescence intensity of Tks5-myc and EEA1 relative to background along a transect (white box in merged image).

The observations made of the GFP-FYVE domain of Hrs were also generally true of the localization pattern of the early endosome marker EEA1 in Src-3T3 cells. EEA1 localizes to punctate structures near the nucleus when cells are transfected with wild-type Tks5-myc, even when invadopodia rosettes remain abundant (Fig 9). This same peri-nuclear localization pattern persisted in Src-3T3 cells regardless of the form of Tks5 being transfected or whether invadopodia were present. The major difference here, as observed previously, was that the m4 and m5 mutant Tks5 constructs remain localized to invadopodia while the m1-m3 mutant Tks5 constructs co-localized exclusively with EEA1 at endosomes (Fig 9).

## Discussion

Tks5 is a scaffolding protein for lipids and proteins and collectively with these biomolecules facilitates invadopodia formation, including the extracellular matrix-remodeling invadopodia activity of invasive cell types. The amino terminal PX domain binds phosphoinositides (*i*.*e*. PtdIns(3)P and PtdIns(3,4)P_2_), places Tks5 at membranes, and is necessary and sufficient for its localization to invadopodia [5]. Also responsible for Tks5 recruitment to invadopodia are the adaptor proteins Nck and Grb2, the former of which utilizes its SH2 domain to bind to a phosphotyrosine in Tks5 (*i*.*e*. pY557) following Src-dependent Tks5 phosphorylation [11], the latter of which utilizes its SH3 domain to bind to one of Tks5’s proline-rich motifs [13]. What remains are a series of five different SH3 domains in Tks5, each varying from 27.5–48% identity [4], each capable of imparting important functionality towards the development of invadopodia and/or any other number of cellular activities to which Tks5 may be involved. It was the relationship between the SH3 domains of Tks5 and invadopodia development that was studied here.

SH3 domains support moderate affinity binding to poly-proline motifs and commonly facilitate binding within and/or between proteins. Using individual SH3 domains as bait has provided some information regarding potential Tks5 binding partners and therefore potential Tks5 functions. In GST-tagged SH3 domain pull down assays, we have previously shown that the 3^rd^ and 5^th^ SH3 domains of Tks5 have broader protein binding spectrums [5]. This is consistent with another group’s mass spectrometry analysis of GST-SH3 domain binding partners that included dynamin (1^st^, 3^rd^, and 5^th^ SH3 domains), N-WASp (all 5 SH3 domains), WIP (3^rd^ and 5^th^ SH3 domains), tubulin (3^rd^ SH3 domain), zyxin (3^rd^ and 5^th^ SH3 domains), nogo-B (5^th^ SH3 domain), and actin (5^th^ SH3 domain) [13], and hint of a possible regulatory role for Tks5 in cytoskeletal dynamics. Other researchers have identified Tks5 SH3 domain interactions through studies of other proteins, including β-dystroglycan (3^rd^ SH3 domain) and XB130 (5^th^ SH3 domain) [16, 22]. Prominent among the clones captured in a phage display assay using the 5^th^ SH3 domain of Tks5 as bait were members of the ADAMs family of metalloproteinases, namely ADAM12, ADAM15, and ADAM19 [5]. These interactions were isolated to proline-rich motifs in the cytoplasmic tail regions of the proteases. Later, the shedding of cell surface HG-EGF by ADAM12 was identified as a key mechanism in the hypoxia-dependent promotion of invadopodia development [15]. As ADAM12 is a Src substrate and ADAM12 phosphorylation aids membrane targeting, it is possible that Src and/or Tks5 facilitate recruitment of ADAM12 in the formation of mature and active invadopodia structures in cancer cells.

Though we have observed weaker binding to the 1^st^ and 2^nd^ SH3 domains of Tks5 before, using these first two SH3 domains in tandem can generate considerably more robust interactions (Salinsky, Seals, and Courtneidge, unpublished data). Such a super SH3 domain module with targets unique to those bound by each SH3 domain individually has been described for Tks5, including the invadopodia marker proteins Sos1 and dynamin [23]. Dynamin, in particular, is well-appreciated for its role in invadopodia development [24]. Like its homolog p47Phox, which has a PX domain followed by two SH3 domains, Tks5 binds to p22Phox through a mechanism that also involves one or both of the first two SH3 domains [25]. This association is linked to the production of reactive oxygen species (ROS), which can be an important element in invadopodia development. If ROS production is inhibited, either by inhibition of the NADPH oxidase system directly or by silencing its subunits, including p22Phox and Tks5, invadopodia fail to form [25].

These aforementioned studies confirm an expected complexity surrounding Tks5’s function in cells. Additional complexity is conferred by the alternative splice sites on either side of first SH3 domain of Tks5, and that multiple promoters give rise to multiple Tks5 isoforms, including ones (Tks5β, Tks5short) that lack the lipid-binding PX domain altogether [4, 9]. There is also a homolog of Tks5 called Tks4 [9, 26]. Though the two proteins share some functional redundancy, their clear structural differences (*e*.*g*. Tks4 has only 4 SH3 domains) likely convey some of their unique effects on cells, including the differential regulation of invadopodia-associated matrix degradation in Src-3T3 cells [9, 26, 27].

In this study, we focused specifically on the PX-domain containing isoform of Tks5 (*i*.*e*. Tks5α) by systematically inactivating conserved tryptophan residues in the SH3 domains that are expected to disable interactions with proline-rich motifs. Specific bioassays for both invadopodia formation and invadopodia-associated matrix degradation activity were then conducted in cells following transfection of these mutant Tks5 constructs. Using the human LNCaP prostate carcinoma cell line, we showed that the 1^st^, 2^nd^, and 3^rd^ SH3 domains of Tks5 were instrumental in regulating gelatin matrix degradation activity. Inactivation of any one of these SH3 domains allowed for a 2.5- to 4.1-fold increase in gelatin degradation over wild-type Tks5. In contrast, in the Src-3T3 cell line, these mutant forms of Tks5, along with Src itself, were mis-localized to what are likely endosome compartments, and were associated with a remarkable loss of invadopodia structures and a greater than 5.0-fold decrease in gelatin-degrading invadopodia activity in the transfected cells.

It is worth considering whether these seemingly disparate results could be explained by the presence of different Tks5 conformational states. One conformation might involve interactions between one or more of the first three SH3 domains of Tks5 with one or more of its own proline-rich motifs. This intramolecular association (or possibly intermolecular Tks5-Tks5 interaction; [11]) would sequester the PX domain and disable its lipid-binding functions. By extension, the phosphorylation of Tks5, by for example Src, would relieve Tks5 of these associations and free both the PX and SH3 domains for new-found lipid- and protein-scaffolding activities. Such a model of Tks5 activation is not without precedent. Typically, p47Phox is held in a closed conformation where its tandem SH3 domains bind to a proline-rich motif embedded within its own PX domain [28]. But in activated cells, there is phosphorylation of serine residues near the carboxy terminus of the protein that releases the intramolecular association such that the PX domain is now free to bind phosphoinositides at the plasma membrane and the tandem SH3 domains are now free to bind p22Phox. This helps build the NADPH oxidase complex necessary for ROS production. Relative to Tks5, the key aspects of this p47Phox activation mechanism are the relatedness of the two proteins to each other, the common presence of a proline-rich motif within their PX domains, and their shared property as kinase substrates.

LNCaP cells have little expression of active Src or Tks5 [12]. Any Tks5 introduced into these cells would be activated only when in close proximity to the basal levels of activated Src and/or PtdIns(3,4)P_2_ located at the plasma membrane, which is where Tks5 phosphorylation, conformational rearrangements, and lipid-binding would likely occur [10]. Once bound to the membrane, Tks5 may then help initiate the low and diffuse levels of gelatin matrix degradation observed in this cell line following transient transfection with Tks5 constructs [12]. In this model, mutations in any of the first three SH3 domains of Tks5 would be expected to unmask the PX domain, thus allowing for increased binding of Tks5 to phosphoinositides at the plasma membrane and the observed increase in matrix degradation. Interestingly, these acute changes in Tks5 expression brought on by transient transfection did not induce significant invadopodia formation in this model system, but our past data with stable Tks5-overexpressing LNCaP cell lines showed punctate invadopodia capable of focalized matrix degradation [12]. It is therefore possible that more extensive and/or more sustained Tks5 expression is necessary to form more mature invadopodia structures in this cell line.

Src-3T3 cells, in contrast, naturally form invadopodia downstream of constitutive Src tyrosine kinase activity and high endogenous Tks5 expression. In this model system, mutations disrupting protein-binding to any of the first three SH3 domains resulted in the accumulation of Tks5 near the nucleus where gelatinolytic activity was notably lacking. Moreover, the co-localization of the m1-m3 Tks5 mutants with the FYVE domain of Hrs and EEA1 suggested that these structures were endosomes. Thus, disrupted binding to the first three SH3 domains of Tks5 might unmask the PX domain and allow for promiscuous binding to phosphoinositide-rich organelles like endosomes, which would be prominent in hyperactivated Src-3T3 cells. Wild-type Tks5 and the m4 and m5 mutant Tks5 constructs, in contrast, localize correctly to invadopodia as they remain inactivated until in close proximity to Src. Once phosphorylated, conformational changes allow for the unfolding of these forms of Tks5 and stabilized binding to the plasma membrane via the PX domain. This provides a mechanism for how Src phosphorylation leads to Tks5 activity at invadopodia. Only more direct studies will discern whether such domain interactions are real and whether they are capable of manipulation in cancerous cells.

Src-3T3 cells are known to create particularly robust invadopodia in the form of ring-shaped super-structures called rosettes. However, expression of Tks5 harboring mutations in any one of the first three SH3 domains had a dominant-negative effect on invadopodia rosette formation. Gelatin matrix degradation was also severely compromised, and the degradation pattern was not as focalized as it was in non-transfected cells, or in cells transfected with the m4 and m5 mutant Tks5 constructs. Moreover, activated Src became co-localized with the m1-m3 mutant forms of Tks5 around the nucleus. It is known that Src is delivered to invadopodia through endosomal trafficking [29]. It is further known that disruptions in trafficking machinery, such as those associated with Rab mutations, result in the retention of Src at endosomes [30]. A study done in MDA-MB-231 breast carcinoma cells also showed that an inhibition of SNARE trafficking machinery, specifically of syntaxin13 and SNAP23, resulted in a decrease in the number of invadopodia formed by these cells as well as in the amount of Src found at the plasma membrane [31]. Also, when observing the focal adhesions of HeLa ovarian carcinoma cells and human foreskin fibroblasts, depletion of the endocytic regulatory protein MICAL-L1 resulted in a loss of active Src at focal adhesions and a decrease in cell migration [32]. These studies suggest that a disruption of endosomal machinery can disrupt the trafficking of Src in cells and thereby impair the ability of cells to form invadopodia, much like what was observed with the Src-3T3 cells transfected with the m1-m3 mutant Tks5 constructs.

There is evidence that Tks5 can bind to endosomal trafficking machinery. This includes the kinesin Eg5, the myosin subunits myosin light polypeptide 6 and myosin regulatory light chain 2, and tubulin [11]. Microtubules provide a mechanical scaffold for protein sorting, while motor proteins allow for movement of vesicular cargo along these cytoskeletal tracks. It is possible that Tks5 plays a role in the motor protein driven vesicle trafficking of Src and other invadopodia marker proteins. Most intriguing is the recent discovery of an interaction between Rab40b and the PX domain of Tks5 [33]. Rab40b functions in the release of MMP2 and MMP9 from secretory vesicles at invadopodia [34]. It seems that these vesicles reach invadopodia with the Tks5 PX domain already bound to phosphoinositides and where Tks5, now acting as an effector of Rab40b, helps tether vesicles to the discrete sites at the plasma membrane where invadopodia form [33]. Perhaps it is the case that when Tks5 is mis-localized, as with the m1-m3 Tks5 mutations, this trafficking system is disrupted and one or more proteins, including Src itself, are unable to be properly delivered, thus resulting in a loss of invadopodia formation and/or function.

In sum, we speculate that Tks5 may occupy different conformational states in the cell where the SH3 domains control accessibility of the PX domain and where phosphorylation by Src activates lipid-binding and the other Tks5 functions associated with invadopodia development. One of these functions may be in the endosomal trafficking of Src to invadopodia. In this way, Tks5 may function both upstream and downstream of Src allowing for a positive feedback loop in invadopodia development and maturation.
